# Acupuncture and Traditional Chinese Herbal Medicine Integrated With Conventional Rehabilitation for Post-stroke Functional Recovery: A Retrospective Cohort Study

**DOI:** 10.3389/fnins.2022.851333

**Published:** 2022-03-16

**Authors:** Cheng-Yu Tseng, Pei-Shan Hsu, Chang-Ti Lee, Hui-Fen Huang, Chou-Chin Lan, Tsung-Han Hsieh, Guan-Ting Liu, Chan-Yen Kuo, Ming-Chieh Wang, Po-Chun Hsieh

**Affiliations:** ^1^Department of Chinese Medicine, Taipei Tzu Chi Hospital, Buddhist Tzu Chi Medical Foundation, New Taipei City, Taiwan; ^2^School of Post-Baccalaureate Chinese Medicine, Tzu Chi University, Hualien, Taiwan; ^3^Division of Pulmonary Medicine, Taipei Tzu Chi Hospital, Buddhist Tzu Chi Medical Foundation, New Taipei City, Taiwan; ^4^School of Medicine, Tzu Chi University, Hualien, Taiwan; ^5^Department of Research, Taipei Tzu Chi Hospital, Buddhist Tzu Chi Medical Foundation, New Taipei City, Taiwan; ^6^Department of Pharmacy, Taipei Tzu Chi Hospital, Buddhist Tzu Chi Medical Foundation, New Taipei City, Taiwan

**Keywords:** acupuncture, traditional Chinese herbal medicine, post-stroke rehabilitation, activities of daily living, integrated traditional Chinese and western medicine

## Abstract

**Background:**

Stroke leads to tremendous impacts on patients and the healthcare system. It is crucial to explore the potential management of rehabilitation. Acupuncture and traditional Chinese herbal medicine (TCHM) integrated with conventional rehabilitation benefit post-stroke functional recovery.

**Methods:**

We retrospectively reviewed the medical records of all patients included in the Integrated Traditional Chinese-Western Medicine care program for stroke (ITCWM-stroke care program) in 2019 in Taipei Tzu Chi Hospital to investigate the effects of acupuncture and TCHM integrated with conventional rehabilitation on National Institutes of Health Stroke Scale (NIHSS) and Barthel Index (BI) scores before and after the program.

**Results:**

A total of 255 stroke inpatients were retrieved and divided into acupuncture and acupuncture + TCHM group by hemorrhagic and ischemic stroke types, respectively. All the patients were recruited in the program at the early subacute phase after stroke onset. Of the hemorrhagic and ischemic stroke subjects, the NIHSS and BI total scores were significantly improved in the acupuncture and acupuncture + TCHM groups. The subgroup analysis results showed that in subjects with a baseline BI score ≤ 40, the acupuncture + TCHM group significantly improved BI total score better than the acupuncture group in both hemorrhagic (*p* < 0.05) and ischemic (*p* < 0.05) stroke subjects.

**Conclusion:**

Acupuncture and TCHM integrated with conventional rehabilitation significantly improve stroke patients’ functional recovery at the early subacute phase. Acupuncture + TCHM contributes to better activities of daily living (ADL) improvements in stroke patients with a baseline BI score ≤ 40. We suggest integrating acupuncture and TCHM into the post-stroke rehabilitation strategy, especially for stroke patients with poor ADL function.

## Introduction

Stroke (including ischemic stroke and hemorrhagic stroke) affects 13.7 million people globally per year and is the second leading cause of death, with 5.5 million deaths per year (GBD 2017 Disease and Injury Incidence and Prevalence Collaborators, 2018; Campbell et al., 2019). Advances in acute stroke management, such as intravenous thrombolytic and endovascular thrombectomy, have significantly increased the post-stroke survival rate in recent years (Powers et al., 2019). However, most stroke patients still suffer from long-term sequelae such as weakness, spasticity, sensory loss, aphasia, dysphagia, depression, and cognitive changes (Powers et al., 2018; Campbell et al., 2019). Stroke causes complex disabilities of activities of daily living (ADL) and health-related quality of life and significantly elevates the socioeconomic burden for stroke survivors, their families, and the healthcare system (Donkor, 2018; GBD 2017 Disease and Injury Incidence and Prevalence Collaborators, 2018). According to the Taiwan Ministry of Health and Welfare, the stroke mortality rate is 43.5 deaths per 100,000 population, which is the fourth highest cause of death in 2019 in Taiwan (The ten leading causes of death in Taiwan, 2019). In Taiwan, the stroke treatment cost is estimated to be 475 million US dollars per year (Hsieh and Chiou, 2014). The average years of potential life lost before 70 years old for stroke is 13.8 years (Hsieh and Chiou, 2014). The disability-adjusted life years (DALYs) are the sum of the years of life lost due to premature mortality (YLLs) and the years lived with a disability (YLDs) due to prevalent cases of the disease or health condition in a population (GBD 2019 Diseases and Injuries Collaborators, 2020). The burden of stroke increased to 1482.03 DALYs per 100,000 population in Taiwan, while 1851.15 DALYs per 100,000 population in the world in 2019 (GBD 2019 Diseases and Injuries Collaborators, 2020). With significant multifaceted impacts on the individual and society, it is essential to expand rehabilitation management for stroke survivors.

Acupuncture is demonstrated to improve global neurological deficiency, specific neurological impairments, and dependency without serious adverse events in stroke patients in the convalescent stage (Yang et al., 2016). Recent meta-analysis studies demonstrate that rehabilitation programs combined with acupuncture can better improve post-stroke cognitive impairment (Hung et al., 2019), dysphagia (Li and Deng, 2019), aphasia (Zhang B. et al., 2019), insomnia (Lee and Lim, 2016), depression (Zhang X.Y. et al., 2019), spasticity (Lim et al., 2015), and shoulder-hand syndrome (Peng et al., 2018). It has also been reported that compared to rehabilitation therapy alone, rehabilitation therapy combined with acupuncture significantly improves limb movement on the Fugl-Meyer Assessment (FMA) scale and ADL performance on the Barthel Index (BI) scale and the Modified Barthel Index (MBI) scale (Peng et al., 2018). Patients with ischemic stroke who received conventional medication and acupuncture showed a lower risk of stroke recurrence in a Taiwanese nationwide retrospective cohort study (Shih et al., 2015).

An overview of Cochrane systematic reviews reveals the potential efficacy of traditional Chinese herbal medicine (TCHM) to improve neurological deficits in stroke patients (Wei et al., 2020). A multicenter randomized controlled trial study demonstrated that conventional rehabilitation combined with acupuncture and TCHM is more effective and safe for functional recovery in subacute stroke patients, under assessment by the MBI, FMA, National Institutes of Health Stroke Scale (NIHSS), Mini-Mental State Examination, Montreal Cognitive Assessment, Hamilton’s Depression Scale, and Self-Rating Depression Scale (Fang et al., 2016). The TCHM prescriptions are also reported to relieve stroke-associated complications and reduce the risk factors and stroke recurrence rate (Chang et al., 2016; Peng et al., 2017). A recent population-based study in Taiwan demonstrated that the number of traditional Chinese medicine (TCM) users among stroke patients increased from 2006 to 2012 (Chen W.S. et al., 2020). In the TCM users among stroke patients, 68.8–79.7% received acupuncture only, while 17.8–26.1% received acupuncture + TCHM (Chen W.S. et al., 2020).

With emerging evidence, the Taiwan National Health Insurance Administration, the Ministry of Health and Welfare established an Integrated Traditional Chinese-Western Medicine care program for stroke (ITCWM-stroke care program) to improve neurological function, ADL, and health-related quality of life (HRQL) of stroke inpatients and decrease the length of stay and medical expenses. After enrolling in the ITCWM-stroke care program, the patients received conventional medical care and rehabilitation integrated with acupuncture and TCHM. We implemented the program in collaboration with rehabilitation physicians, neurologists, and neurosurgeons in Taipei Tzu Chi Hospital. We conducted a retrospective cohort analysis to investigate the effects of acupuncture and TCHM integrated with conventional rehabilitation in post-stroke management.

## Materials and Methods

### Ethics Approval and Consent to Participate

The study protocols and procedures were approved by the Research Ethics Committee of Taipei Tzu Chi Hospital, Buddhist Tzu Chi Medical Foundation (No. 09-X-049), which waived the need for patient informed consent since the study utilized secondary de-identified information.

### Participants and Data Collection

Stroke inpatients were assessed by rehabilitation physicians, neurologists, and neurosurgeons in Taipei Tzu Chi Hospital. The inclusion criteria of the ITCWM stroke care program were as follows: (1) age > 18 years (2) stroke as defined by the ICD-10 classification (G45, G46, I60, I61, I62, I63, I65, I66, I67, I68); (3) within six months after the first stroke onset; and (4) stable condition. The exclusion criteria were as follows: (1) traumatic brain injury, (2) brain tumor, (3) transient ischemic attack, (4) hemorrhagic transformation of ischemic stroke, (5) certifiable infectious disease or rare disease, and (6) pregnant woman. TCM physicians were then consulted for the ITCWM-stroke care program if the inclusion criteria were met. After enrollment in the ITCWM-stroke care program, the stroke patients received conventional medical care and rehabilitation therapies (including physical therapy, occupational therapy, and speech therapy), combined with personalized treatments by acupuncture (retained for 20 min three times a week, on every Monday, Wednesday, and Friday) and TCHM. We used scalp acupuncture with CV20, EX-HN1 (Sishencong), anterior and posterior oblique line of vertex-temporal as primary acupoints, accompanied with empirical acupoints used for various complications. We prescribed TCHM mainly for clear heat and purge fire, resolve phlegm to open the orifices, calm the liver to extinguish wind, dispel wind to free the collateral vessels, and soothe the liver and regulate qi, accompanied with empirical TCHM based on TCM diagnostic patterns. ITCWM-stroke care continued until the patient was discharged or suspended due to the patient’s unstable condition. The outcome variables measured in this study include NIHSS and BI. Outcome measurement scores were assessed before and after the ITCWM-stroke care program.

**FIGURE 1:**
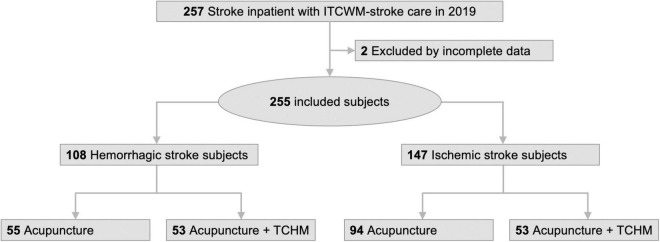
Study flow diagram.

We retrospectively reviewed the medical records of all patients included in the ITCWM-stroke care program in 2019. We also used the AICS LUMOS (ASUS AICS, 2020) electronic medical record search system for data investigation. We collected the following information for each patient: stroke type, age, sex, underlying disease, the period after stroke onset to ITCWM-stroke care, total treatment course, and time of ITCWM-stroke care, TCHM prescription, severe adverse effects, NIHSS, and BI scores. The retrieved subjects were divided into acupuncture group and acupuncture + TCHM group by hemorrhagic and ischemic stroke types to compare the clinical effects on NIHSS and BI scores (Figure 1).

### National Institutes of Health Stroke Scale

The NIHSS is a 15-item scale that standardizes and quantifies the basic neurological examination to objectively quantify the impairment primarily caused by stroke. The NIHSS items are as follows: 1a [Level of Consciousness (LOC) Responsiveness], 1b (LOC Questions), 1c (LOC Commands), 2 (Horizontal Eye Movement), 3 (Visual field test), 4 (Facial Palsy), 5aL (Motor Left Arm), 5bR (Motor Right Arm), 6aL (Motor Left Leg), 6bR (Motor Right Leg), 7 (Limb Ataxia), 8 (Sensory), 9 (Language), 10 (Speech), 11 (Extinction and Inattention). NIHSS scored from 0 (no impairment) to a maximum of 42 (total impairment) (Brott et al., 1989). It has been suggested that a change in the NIHSS of more than two points represents clinically relevant early improvement or deterioration (Adams et al., 1999).

### Barthel Index

The BI is the most commonly used functional measure in stroke-rehabilitation settings (Harrison et al., 2013). The BI assesses ten functional domains of ADL, scoring the individual depending on independence in each domain. Scores range from 0 and 100, with a higher score indicating greater independence (Harrison et al., 2013). The BI domains are as follows: 1 (Feeding), 2 (Bathing), 3 (Grooming), 4 (Dressing), 5 (Bowels), 6 (Bladder), 7 (Toilet use), 8 [Transfers (bed to chair and back)], 9 [Mobility (on level surface)], and 10 (Stairs). It is suggested that patients with BI > 80 are generally independent, while patients with BI < 40 are very dependent (Harrison et al., 2013).

### Statistical Analysis

All statistical analyses were conducted using GraphPad Prism 9 for macOS (Version 9.2.0, GraphPad Software, San Diego, CA, United States^1^). Baseline demographic and clinical characteristics are presented as patient number (%) and mean ± standard deviation (SD). Continuous variables were examined by the independent *t*-test. Categorical variables were analyzed by Fisher’s exact test and the Chi-square test. Outcome measurements are presented as the mean ± SD.

## Results

### Cohort Characteristics

A flow diagram of the study is presented in Figure 1. A total of 257 stroke inpatients who met the criteria finished the ITCWM-stroke care program in Taipei Tzu Chi Hospital in 2019. After retrospectively reviewing the medical records, two patients were excluded due to incomplete data. Overall, 255 stroke subjects (108 hemorrhagic stroke subjects and 147 ischemic stroke subjects) were included and divided into the acupuncture group and acupuncture + TCHM group for analysis, respectively.

The demographic and clinical characteristics of the patients are presented in Table 1. There were no statistically significant differences between the acupuncture group and the acupuncture + TCHM groups with respect to age, sex, baseline clinical characteristics (stroke severity, NIHSS score, BI score, underlying disease), and the period after stroke onset to ITCWM-stroke care. Of the 108 hemorrhagic stroke subjects, 55 subjects (23 female and 32 male) in the acupuncture group with a mean age of 61.71 ± 14.73 years received ITCWM-stroke care 49.71 ± 38.85 days after stroke onset (at early subacute phase, 7 days to 3 months from stroke onset, which is a critical time for neural plasticity and recovery) (Bernhardt et al., 2017). 53 subjects (13 female and 40 male) in acupuncture + TCHM group with mean age 57.91 ± 15.03 years, received ITCWM-stroke care 62.42 ± 35.73 days after stroke onset (at early subacute phase). Of the 147 ischemic stroke subjects, 94 subjects (34 female and 60 male) in the acupuncture group with a mean age of 69.40 ± 12.04 years received ITCWM-stroke care 26.06 ± 27.57 days after stroke onset (at early subacute phase); 53 subjects (26 female and 27 male) in acupuncture + TCHM group with a mean age of 69.13 ± 11.35 years received ITCWM-stroke care 35.30 ± 37.64 days after stroke onset (at early subacute phase). No severe adverse effects were recorded.

**TABLE 1:** Baseline demographic and clinical characteristics.

Variables	Hemorrhagic stroke			Ischemic stroke		
	Acupuncture	Acupuncture + TCHM	*p* value	Acupuncture	Acupuncture + TCHM	*p* value
Patient number, n	55	53		94	53	
**Gender, n (%)**						
Female	23 (41.82%)	13 (24.53%)	0.0679	34 (36.17%)	26 (49.06%)	0.1622
Male	32 (58.18%)	40 (75.47%)		60 (63.83%)	27 (50.94%)	
Age, mean ± SD	61.71 ± 14.73	57.91 ± 15.03	0.1870	69.40 ± 12.04	69.13 ± 11.35	0.8933
**Stroke severity, n (%)**						
Mild (NIHSS: ≤7)	14 (25.45%)	14 (26.42%)	0.1310	48 (51.06%)	20 (37.74%)	0.1937
Moderate (NIHSS: 8-16)	16 (29.09%)	24 (45.28%)		29 (30.85%)	24 (45.28%)	
Severe (NIHSS: ≥17)	25 (45.45%)	15 (28.30%)		17 (18.09%)	9 (16.98%)	
NIHSS (total), mean ± SD	18.24 ± 15.25	13.45 ± 10.41	0.1391	11.47 ± 12.56	10.13 ± 5.27	0.1813
Barthel Index (total), mean ± SD	24.72 ± 30.30	27.31 ± 27.75	0.2035	35.48 ± 32.20	32.55 ± 25.09	0.9782
**Underlying disease, n (%)**						
Hypertension	49 (90.74%)	45 (86.54%)	0.4875	77 (81.91%)	43 (81.13%)	0.6477
Diabetes mellitus	10 (18.52%)	12 (22.64%)		42 (44.68%)	18 (33.96%)	
Hyperlipidemia	5 (9.26%)	2 (3.77%)		37 (39.36%)	16 (30.19%)	
Stroke onset to first ITCWM-stroke care, day	49.71 ± 38.85	62.42 ± 35.73	0.0630	26.06 ± 27.57	35.30 ± 37.64	0.5320

### Changes in National Institutes of Health Stroke Scale and Barthel Index Scores of Hemorrhagic Stroke Subjects

The changes in NIHSS and BI scores of the hemorrhagic stroke subjects before and after the ITCWM-stroke care program are presented in Table 2.

**TABLE 2:** NIHSS and Barthel Index scores in hemorrhagic stroke patients with ITCWM-stroke care.

Hemorrhagic stroke						
	Acupuncture (*n* = 55)			Acupuncture + TCHM (*n* = 53)		
NIHSS items	Before	After	*p* value	Before	After	*p* value
1a (LOC Responsiveness)	0.78 ± 1.05	0.62 ± 0.95	0.0312*	0.34 ± 0.65	0.17 ± 0.51	0.0078*
1b (LOC Questions)	1.00 ± 0.96	0.82 ± 0.94	0.0156*	0.87 ± 0.94	0.66 ± 0.88	0.0020*
1c (LOC Commands)	0.75 ± 0.93	0.65 ± 0.91	0.1875	0.47 ± 0.77	0.26 ± 0.62	0.0078*
2 (Horizontal Eye Movement)	0.36 ± 0.70	0.35 ± 0.70	>0.9999	0.21 ± 0.49	0.11 ± 0.38	0.1250
3 (Visual field test)	0.40 ± 0.93	0.36 ± 0.91	>0.9999	0.28 ± 0.60	0.15 ± 0.46	0.0312*
4 (Facial Palsy)	0.78 ± 0.90	0.65 ± 0.82	0.0312*	0.57 ± 0.75	0.43 ± 0.67	0.0156*
5aL (Motor Left Arm)	2.16 ± 2.48	2.33 ± 2.76	0.5391	2.08 ± 2.16	1.75 ± 1.84	0.0098*
5bR (Motor Right Arm)	2.27 ± 2.32	2.35 ± 2.62	>0.9999	1.58 ± 2.17	1.21 ± 1.88	0.0010*
6aL (Motor Left Leg)	2.13 ± 2.48	2.24 ± 2.77	0.8652	1.96 ± 2.01	1.72 ± 1.76	0.0176*
6bR (Motor Right Leg)	2.22 ± 2.29	2.22 ± 2.62	0.4771	1.38 ± 1.81	1.11 ± 1.80	0.0005*
7 (Limb Ataxia)	0.31 ± 0.66	0.25 ± 0.62	0.2500	0.15 ± 0.46	0.08 ± 0.33	0.1250
8 (Sensory)	0.73 ± 0.71	0.73 ± 0.76	0.8125	0.66 ± 0.59	0.58 ± 0.57	0.1250
9 (Language)	1.38 ± 1.24	1.25 ± 1.22	0.1250	0.70 ± 0.97	0.58 ± 0.82	0.0625
10 (Speech)	2.84 ± 3.53	2.71 ± 3.59	0.2969	1.85 ± 3.13	1.53 ± 3.00	0.0459*
11 (Extinction and Inattention)	0.29 ± 0.63	0.24 ± 0.58	0.3750	0.40 ± 0.66	0.25 ± 0.52	0.0156*
Total	18.24 ± 15.25	17.76 ± 16.94	0.0017*	13.45 ± 10.41	10.60 ± 9.66	<0.0001*

**Barthel Index domains**	**Before**	**After**	***p* value**	**Before**	**After**	***p* value**

1 (Feeding)	3.15 ± 3.92	3.89 ± 4.20	0.0078*	4.33 ± 4.54	5.58 ± 4.50	0.0039*
2 (Bathing)	1.20 ± 2.16	1.48 ± 2.30	0.2500	1.35 ± 2.24	1.83 ± 2.43	0.0625
3 (Grooming)	1.85 ± 3.26	2.50 ± 3.33	0.0156*	1.92 ± 3.16	3.17 ± 3.43	0.0005*
4 (Dressing)	0.28 ± 1.16	0.46 ± 1.46	0.5000	0.38 ± 1.35	0.48 ± 1.49	>0.9999
5 (Bowels)	1.94 ± 3.28	2.22 ± 3.46	0.2500	1.83 ± 3.14	2.69 ± 3.35	0.0078*
6 (Bladder)	4.35 ± 4.66	5.00 ± 4.56	0.0156*	6.35 ± 3.72	7.50 ± 3.50	0.0005*
7 (Toilet use)	4.44 ± 4.62	4.72 ± 4.60	0.2500	5.29 ± 4.47	6.44 ± 4.00	0.0020*
8 [Transfers (bed to chair and back)]	3.06 ± 5.36	3.98 ± 5.94	0.0312*	2.21 ± 4.13	3.94 ± 5.36	0.0010*
9 (Mobility [on level surface])	1.02 ± 2.64	1.67 ± 3.36	0.0312*	0.96 ± 2.81	1.44 ± 3.18	0.1250
10 (Stairs)	3.43 ± 5.03	4.44 ± 5.38	0.0020*	2.69 ± 4.69	4.33 ± 5.60	0.0010*
Total	24.72 ± 30.30	30.00 ± 32.42	<0.0001*	27.31 ± 27.75	37.31 ± 29.08	<0.0001*

*p < 0.05, NIHSS, National Institutes of Health Stroke Scale; TCHM: traditional Chinese herbal medicine.

In the acupuncture group (*n* = 55), the subjects were treated with ITCWM-stroke care for a total of 7.65 ± 4.71 courses (within 22.49 ± 12.01 days). All the NIHSS items improved after the ITCWM-stroke care program. The total NIHSS score was 18.24 ± 15.25 at enrollment and improved to 17.76 ± 16.94 at discharge (*p* = 0.0017). The differences in items 1a, 1b, 4, and the total NIHSS score were statistically significant. All the BI domains were also improved after the ITCWM-stroke care program. The total BI score was 24.72 ± 30.30 at enrollment and improved to 30.00 ± 32.42 at discharge (*p* < 0.0001). The differences in domains 1, 3, 6, 8, 9, 10, and total BI scores were statistically significant.

In the acupuncture + TCHM group (*n* = 53), the subjects were treated with ITCWM-stroke care for a total of 9.49 ± 5.33 courses (within 24.38 ± 13.26 days). All the NIHSS items improved after the ITCWM-stroke care program. The total NIHSS score was 13.45 ± 10.41 at enrollment and improved to 10.60 ± 9.66 at discharge (*p* < 0.0001). The differences in items 1a, 1b, 1c, 3, 4, 5aL, 5bR, 6aL, 6bR, 10, 11, and total NIHSS scores were statistically significant. All the BI domains were also improved after the ITCWM-stroke care program. The total BI score was 27.31 ± 27.75 at enrollment and improved to 37.31 ± 29.08 at discharge (*p* < 0.0001). The differences in domains 1, 3, 5, 6, 7, 8, 10, and the total BI score were statistically significant.

### Changes in National Institutes of Health Stroke Scale and Barthel Index Scores of Ischemic Stroke Subjects

The changes of NIHSS and BI scores of the ischemic stroke subjects before and after the ITCWM-stroke care program are presented in Table 3.

**TABLE 3:** NIHSS and Barthel Index scores in ischemic stroke patients with ITCWM-stroke care.

Ischemic stroke						
	Acupuncture (*n* = 94)			Acupuncture + TCHM (*n* = 53)		
NIHSS items	Before	After	*p* value	Before	After	*p* value
1a (LOC Responsiveness)	0.39 ± 1.11	0.32 ± 1.08	0.0391*	0.19 ± 0.52	0.08 ± 0.27	0.1250
1b (LOC Questions)	0.64 ± 1.21	0.54 ± 1.18	0.0449*	0.43 ± 0.77	0.40 ± 0.74	0.7500
1c (LOC Commands)	0.46 ± 1.14	0.40 ± 1.12	0.1875	0.38 ± 0.71	0.25 ± 0.55	0.0625
2 (Horizontal Eye Movement)	0.28 ± 0.59	0.24 ± 0.56	0.5000	0.09 ± 0.30	0.08 ± 0.27	>0.9999
3 (Visual field test)	0.28 ± 0.72	0.20 ± 0.63	0.1250	0.23 ± 0.61	0.13 ± 0.44	0.1250
4 (Facial Palsy)	0.70 ± 0.91	0.57 ± 0.85	0.0032*	0.53 ± 0.61	0.43 ± 0.54	0.1797
5aL (Motor Left Arm)	1.40 ± 1.96	1.32 ± 1.96	0.1357	1.83 ± 1.73	1.51 ± 1.65	0.0186*
5bR (Motor Right Arm)	1.52 ± 2.05	1.43 ± 2.02	0.0122*	1.13 ± 1.52	0.98 ± 1.50	0.2129
6aL (Motor Left Leg)	1.29 ± 1.92	1.26 ± 1.96	0.6482	1.57 ± 1.47	1.23 ± 1.32	0.0002*
6bR (Motor Right Leg)	1.31 ± 1.90	1.26 ± 1.91	0.0625	0.92 ± 1.28	0.75 ± 1.22	0.0938
7 (Limb Ataxia)	0.39 ± 0.71	0.32 ± 0.64	0.1250	0.32 ± 0.67	0.23 ± 0.58	0.2500
8 (Sensory)	0.48 ± 0.58	0.43 ± 0.56	0.1250	0.47 ± 0.50	0.42 ± 0.50	0.5078
9 (Language)	0.73 ± 0.97	0.63 ± 0.94	0.0039*	0.60 ± 0.84	0.57 ± 0.89	0.5312
10 (Speech)	1.53 ± 2.81	1.17 ± 2.47	0.0007*	1.28 ± 2.32	1.09 ± 2.07	0.3750
11 (Extinction and Inattention)	0.34 ± 0.66	0.27 ± 0.61	0.0312*	0.13 ± 0.34	0.06 ± 0.23	0.1250
Total	11.47 ± 12.56	10.06 ± 12.26	<0.0001*	10.13 ± 5.27	8.21 ± 4.76	<0.0001*

**Barthel Index domains**	**Before**	**After**	***p* value**	**Before**	**After**	***p* value**

1 (Feeding)	4.89 ± 4.46	5.59 ± 4.39	0.0010*	5.66 ± 4.05	6.51 ± 3.74	0.0312*
2 (Bathing)	1.76 ± 2.40	2.02 ± 2.47	0.0625	1.70 ± 2.39	2.08 ± 2.49	0.1250
3 (Grooming)	3.09 ± 3.75	3.78 ± 3.86	0.0002*	2.36 ± 3.04	3.30 ± 3.39	0.0063*
4 (Dressing)	0.69 ± 1.74	0.74 ± 1.79	>0.9999	0.19 ± 0.96	0.38 ± 1.33	0.5000
5 (Bowels)	2.82 ± 3.71	3.62 ± 3.84	0.0001*	2.36 ± 3.04	3.02 ± 3.30	0.0391*
6 (Bladder)	6.12 ± 4.41	6.97 ± 4.10	0.0002*	7.17 ± 3.86	7.74 ± 3.48	0.2500
7 (Toilet use)	5.90 ± 4.46	6.44 ± 4.38	0.0078*	6.60 ± 4.13	7.74 ± 3.48	0.0039*
8 [Transfers (bed to chair and back)]	3.78 ± 5.52	5.05 ± 5.87	<0.0001*	2.74 ± 5.05	4.72 ± 6.00	0.0011*
9 [Mobility (on level surface)]	1.49 ± 3.01	2.13 ± 3.63	0.0005*	0.75 ± 2.06	1.42 ± 3.16	0.0312*
10 (Stairs)	4.95 ± 5.89	6.12 ± 6.05	<0.0001*	3.02 ± 4.94	4.62 ± 5.45	0.0002*
Total	35.48 ± 32.20	42.45 ± 33.28	<0.0001*	32.55 ± 25.09	41.98 ± 26.46	<0.0001*

*p < 0.05, NIHSS, National Institutes of Health Stroke Scale; TCHM, traditional Chinese herbal medicine.

In the acupuncture group (*n* = 94), the subjects were treated with ITCWM-stroke care for a total of 6.66 ± 4.29 courses (within 19.65 ± 12.73 days). All the NIHSS items improved after the ITCWM-stroke care program. The mean total NIHSS score was 11.47 ± 12.56 at enrollment and improved to 10.06 ± 12.26 (*p* < 0.0001). The differences in items 1a, 1b, 4, 5bR, 9, 10, 11, and total NIHSS scores were statistically significant. All the BI domains were also improved after the ITCWM-stroke care program. The mean total BI score was 35.48 ± 32.20 at enrollment and improved to 42.45 ± 33.28 (*p* < 0.0001). The differences in domains 1, 3, 5, 6, 7, 8, 9, 10, and the total BI scores were statistically significant.

In the acupuncture + TCHM group (*n* = 53), the subjects were treated with ITCWM-stroke care for a total of 9.64 ± 3.56 courses (within 27.58 ± 14.66 days). All the NIHSS items improved after the ITCWM-stroke care program. The mean total NIHSS score was 10.13 ± 5.27 at enrollment and improved to 8.21 ± 4.76 (*p* < 0.0001). The differences in items 5aL and 6aL and the total NIHSS scores were statistically significant. All the BI domains were also improved after the ITCWM-stroke care program. The mean total BI score was 32.55 ± 25.09 before and improved to 41.98 ± 26.46 (*p* < 0.0001). The differences in domains 1, 3, 5, 7, 8, 9, 10, and the total BI scores were statistically significant.

### Acupuncture + Traditional Chinese Herbal Medicine Contributes to Better Functional Recovery Than Acupuncture in Stroke Subjects With Baseline Barthel Index Score ≤ 40

**FIGURE 2:**
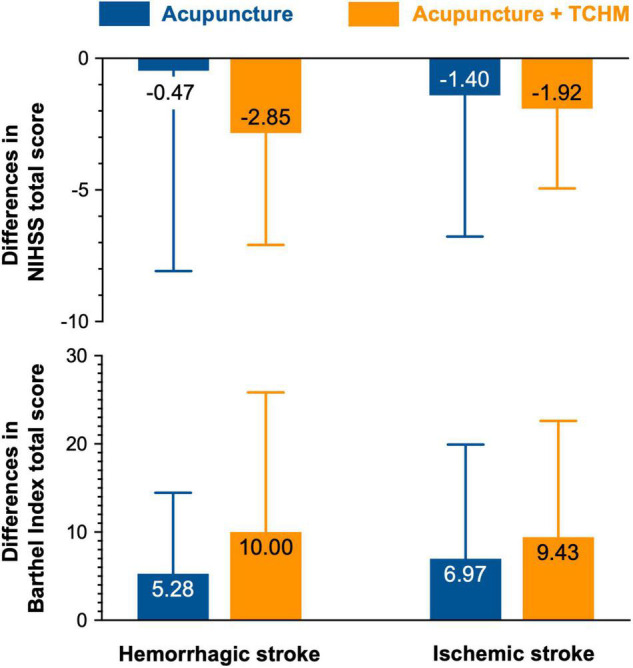
Differences in NIHSS and BI total scores.

The differences in each group’s total NIHSS and BI scores are presented in Figure 2. A decrease of more than two points in the NIHSS score is suggested to represent a clinically relevant improvement (Adams et al., 1999; Harrison et al., 2013). Within a stroke group, an increase in mean BI score by 1.85 points met minimal clinically important difference (Hsieh et al., 2007). Of hemorrhagic stroke subjects, acupuncture improved −0.47 ± 7.61 total NIHSS score, while acupuncture + TCHM improves −2.85 ± 4.24 (*p* = 0.0962). Acupuncture improved 5.28 ± 9.19 total BI score, while acupuncture + TCHM improves 10.00 ± 15.84 total BI score (*p* = 0.0820). Of ischemic stroke subjects, acupuncture improved −1.40 ± 5.37 total NIHSS score, while acupuncture + TCHM improved −1.92 ± 3.03 (*p* = 0.1682). Acupuncture improved 6.97 ± 12.96 total BI score, while acupuncture + TCHM improved 9.43 ± 13.28 total BI score (*p* = 0.0761). The results show that acupuncture + TCHM contributes to better functional recovery than acupuncture. However, there was no statistically significant difference between the acupuncture group and the acupuncture + TCHM group.

**FIGURE 3:**
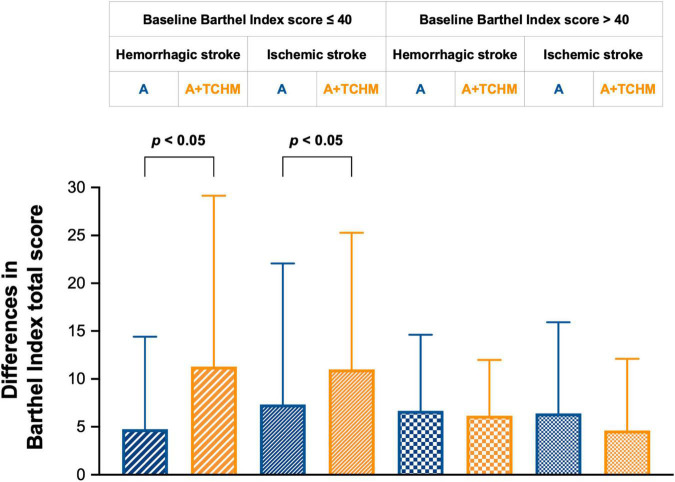
Subgroup analysis by baseline BI score 40.

Patients with BI scores ≤ 40 are very dependent within very basic skills, such as feeding, grooming, and sphincter control (Granger et al., 1979; Harrison et al., 2013). Hence, we conducted a subgroup analysis by baseline BI score ≤ 40 and > 40, according to previous studies (Nakao et al., 2010; Tang et al., 2010). The subgroup analysis results are presented in Figure 3. Of subjects with baseline BI score ≤ 40, in hemorrhagic stroke subjects, acupuncture improved 4.74 ± 9.66 BI score, while acupuncture + TCHM improved 11.28 ± 17.87 BI score (*p* = 0.0436); in ischemic stroke subjects, acupuncture improves 7.33 ± 14.76 BI score, while acupuncture + TCHM improves 11.00 ± 14.29 BI score (*p* = 0.0333). The results demonstrate that in stroke subjects with a baseline BI score ≤ 40, acupuncture + TCHM contributes to better functional recovery than acupuncture with statistical and clinical significance. Of subjects with baseline BI score > 40, in hemorrhagic stroke subjects, acupuncture improved 6.67 ± 7.94 BI score, while acupuncture + TCHM improved 6.15 ± 5.83 BI score (*p* = 0.9043); in ischemic stroke subjects, acupuncture improved 6.39 ± 9.53 BI score, while acupuncture + TCHM improved 4.62 ± 7.49 BI score (*p* = 0.8091). The results demonstrate that in stroke subjects with baseline BI score > 40, acupuncture + TCHM and acupuncture contribute to even functional recovery with clinical significance.

### Traditional Chinese Herbal Medicine Used in the Stroke Subjects

Lonicerae Japonicae Flos (Jin Yin Hua), Pinelliae Rhizoma (Ban Xia), Acori Tatarinowii Rhizoma (Shi Chang Pu), Rhei Radix et Rhizoma (Da Huang), Paeoniae Alba Radix (Bai Shao), Cinnamomi Ramulus (Gui Zhi), Polygonati Odorati Rhizoma (Yu Zhu), Fructus Amomi (Sha Ren), Poria (Fu Ling), and Polygalae Radix (Yuan Zhi) were the ten most prescribed TCHMs in hemorrhagic stroke subjects.

Lonicerae Japonicae Flos (Jin Yin Hua), Pinelliae Rhizoma (Ban Xia), Rhei Radix et Rhizoma (Da Huang), Tatarinowii Rhizoma (Shi Chang Pu), Polygonati Odorati Rhizoma (Yu Zhu), Paeoniae Alba Radix (Bai Shao), Cinnamomi Ramulus (Gui Zhi), Poria (Fu Ling), Fructus Amomi (Sha Ren), and Panacis Quinquefolii Radix (Fen Guang Shen) were the ten most prescribed TCHMs in ischemic stroke subjects.

## Discussion

The results indicate that acupuncture and TCHM significantly improve stroke patients’ functional recovery at the early subacute phase. Furthermore, acupuncture + TCHM results in better ADL improvements than acupuncture in stroke patients with a baseline BI score ≤ 40. This study demonstrates evidence-based recommendations for ITCWM care for stroke patients based on stroke patients’ BI score.

Patients with BI scores ≤ 40 are very dependent (Granger et al., 1979; Harrison et al., 2013). Nakao et al. (2010) demonstrated two recovery paths for patients with a BI score ≤ 40, two-thirds have a significant improvement, while one-third have no change at 6 months. Our result revealed that in subjects with baseline BI score ≤ 40, acupuncture + TCHM improved 11.28 ± 17.87 BI score in hemorrhagic stroke subjects; acupuncture + TCHM improved 11.00 ± 14.29 BI score in ischemic stroke subjects. The improvements were better than the acupuncture groups. We suggested acupuncture + TCHM integrated with conventional rehabilitation for post-stroke patients at the early subacute phase for better prognosis.

The current understanding of the post-stroke brain repair process suggests that major behavioral recovery and rapid neurological changes occur during the first weeks 3 months after stroke (early subacute phase), which is a critical time for neural plasticity (Bernhardt et al., 2017). It is recommended to start the recovery and rehabilitation program at this time (Bernhardt et al., 2017). A previous retrospective cohort study demonstrated that earlier intervention by acupuncture might exert better long-term effects (during the 1-year follow-up) on motor dysfunction and inflammation in treating acute ischemic stroke (Xu et al., 2020). Acupuncture within 2 days is suggested as the optimal treatment timing for early recovery on the 90th day (Xu et al., 2020). For hemorrhagic stroke subjects, we started the ITCWM-stroke care program 49.71 ± 38.85 (acupuncture group) and 62.42 ± 35.73 (acupuncture + TCHM group) days after stroke onset. For ischemic stroke subjects, we started the ITCWM-stroke care program 26.06 ± 27.57 (acupuncture group) and 35.30 ± 37.64 (acupuncture + TCHM group) days after stroke onset. The hemorrhagic stroke patients usually have relatively unstable conditions and are hospitalized in intensive care units in the acute phase, who are not included in the ITCWM-stroke program till the patients’ condition becomes stable. We propose to monitor the stroke survivors’ suitability closer to implementing ITCWM-stroke care as early as possible in the future.

A recent meta-analysis study demonstrated that Paeoniae Rubra Radix (Chi Shao), Angelicae Sinensis Radix (Dang Gui), Astragali Radix (Huang Qi), Puerariae Lobatae Radix (Ge Gen), and Salviae Mihiorrhizae Radix et Rhizoma (Dan Shen) could relieve the symptoms of convalescent stroke patients, promote neurological function recovery, and improve the ADL effectively (Zhang et al., 2020). Many Chinese herbs or herbal formulations (such as Salviae Mihiorrhizae Radix et Rhizoma (Dan Shen), Notoginseng Radix et Rhizoma (San Qi), Astragali Radix (Huang Qi), Bu Yang Huan Wu Tang, and Dang Gui Shao Yao San) have demonstrated significant pro-angiogenic and neuroprotective effects (Seto et al., 2016). Experimental evidence with *in vitro* and *in vivo* settings show that many active compounds or extracts from TCHM can regulate proliferation, self-renewal, and differentiation of neural progenitor cells, promoting neural network formation and neurological functional recovery (Chen et al., 2019). Chrysophanol [extracted from Rhei Radix et Rhizoma (Da Huang)] shows significant neuroprotective and anti-inflammatory effects against ischemic/reperfusion brain injury by inhibiting endoplasmic reticulum stress (Zhao et al., 2018). Asarones α and β [extracted from Acori Tatarinowii Rhizoma (Shi Chang Pu)] promote neurogenesis and hippocampal neural progenitor cell proliferation and improve recognition memory (Chen et al., 2019).

According to a nationwide population-based study in Taiwan, TCHM users have a lower mortality rate than non-TCHM users (Chang et al., 2016). Bu Yang Huan Wu Tang and Salviae Mihiorrhizae Radix et Rhizoma (Dan Shen) are the most commonly prescribed Chinese herbal formulae and single herbs (Chang et al., 2016). Ginkgo biloba extract, Dan Shen agents, Sanchi agents, Chuanxiong, Acanthopanax, Mailuoning, Dengzhanhua, and Tongxinluo capsule were reported with potential efficacy improving neurological deficits in stroke patients (Wei et al., 2020). As for our results, we used many of the TCHM with neuroprotective and neurogenesis effects, such as Acori Tatarinowii Rhizoma (Shi Chang Pu), Rhei Radix et Rhizoma (Da Huang), and Polygalae Radix (Yuan Zhi). We also used TCHM for resolving phlegm to open the orifices, such as Acori Tatarinowii Rhizoma (Shi Chang Pu), Polygalae Radix (Yuan Zhi), Pinelliae Rhizoma (Ban Xia), Fructus Amomi (Sha Ren), and Poria (Fu Ling). Since stroke survivors usually take anticoagulants, we avoid using TCHM, which may increase potential bleeding risk. Our results show that acupuncture + TCHM induces better improvement in BI scores in hemorrhagic and ischemic stroke subjects with a baseline BI score of ≤ 40. The pharmacological effects of TCHM may contribute to enhancing neurological plasticity and repair in patients with worse conditions.

Many underlying mechanisms in treating stroke by acupuncture have been revealed. Promoting neurogenesis and cell proliferation in the central nervous system, regulating cerebral blood flow in the ischemic area, anti-apoptosis in the ischemic area, regulating neurochemicals, and improving impaired long-term potentiation and memory after stroke have been reported (Chavez et al., 2017). Previous studies also suggest that acupuncture enhances stroke recovery by targeting neurogenesis, with increased expression of neurogenesis markers: BrdU, Nestin (proliferation), PSAN-CAM (migration), and NeuN (differentiation), in experimental ischemic stroke animal models (Lu et al., 2016). A previous functional magnetic resonance imaging study showed that acupuncture at the right side of Quchi (LI 11) and Zusanli (ST 36) could stimulate the right precentral gyrus and left supplementary motor, which is possibly related to motor recovery from stroke (Chen S.Q. et al., 2020). Acupuncture has also been reported to modulate the patients’ disrupted patterns of the whole-brain network after the subcortical ischemic stroke (Han et al., 2020).

### Limitation of the Study

This study has some limitations to this study. First, we analyzed the general functional recovery effects of ITCWM-stroke care on patients with any stroke severity. The included patients’ baseline conditions are varied, which may have affected the outcomes. Second, we focused on functional recovery and did not measure cognition, depression, and insomnia with other assessment tools to investigate the effects of the ITCWM-stroke care program on the mental condition. Third, the follow-up period was confined to hospitalization. However, the time-sequence and long-term effects are unavailable. In summary, the elements above could limit the inference of our analysis. Further studies with more rigorous designs, larger patient numbers, and a long-term follow-up period are needed.

## Conclusion

Acupuncture and TCHM integrated with conventional rehabilitation significantly improve stroke patients’ functional recovery at the early subacute phase. Acupuncture + TCHM contributes better ADL improvements in stroke patients with a baseline BI score ≤ 40. We suggest integrating acupuncture and TCHM into post-stroke rehabilitation strategy, especially for stroke patients with poor ADL function.

## Data Availability Statement

The original contributions presented in the study are included in the article/supplementary material, further inquiries can be directed to the corresponding author.

## Ethics Statement

The studies involving human participants were reviewed and approved by the Research Ethics Committee of Taipei Tzu Chi Hospital, Buddhist Tzu Chi Medical Foundation. Written informed consent for participation was not required for this study in accordance with the national legislation and the institutional requirements.

## Author Contributions

P-CH, P-SH, C-TL, and H-FH: study conception and design. C-YT, P-SH, C-TL, and P-CH: data collection. C-YT, P-CH, T-HH, and G-TL: statistical analysis. P-CH, C-YK, M-CW, and C-CL: interpretation of results. C-YT and P-CH: drafting manuscript. P-CH: project administration. All authors reviewed the results and approved the final version of the manuscript.

## Conflict of Interest

The authors declare that the research was conducted in the absence of any commercial or financial relationships that could be construed as a potential conflict of interest.

## Publisher’s Note

All claims expressed in this article are solely those of the authors and do not necessarily represent those of their affiliated organizations, or those of the publisher, the editors and the reviewers. Any product that may be evaluated in this article, or claim that may be made by its manufacturer, is not guaranteed or endorsed by the publisher.

## Footnotes

1
www.graphpad.com
